# A review on the induction of host immunity by the current COVID-19 vaccines and a brief non-pharmaceutical intervention to mitigate the pandemic

**DOI:** 10.1186/s42269-022-00719-x

**Published:** 2022-02-16

**Authors:** Rashed Noor

**Affiliations:** Department of Life Sciences (DLS), School of Environment and Life Sciences (SELS), Independent University, Bangladesh (IUB), Plot 16, Block B, Bashundhara, Dhaka 1229 Bangladesh

**Keywords:** SARS-CoV-2, COVID-19 pandemic, Second wave, Vaccines, SARS-CoV-2 variants, Host immunity, Non-pharmaceutical interventions

## Abstract

**Background:**

To mitigate the current COVID-19 pandemic by the severe acute respiratory coronavirus 2 (SARS-CoV-2), designing of repurposed antiviral drugs and the development of vaccines using different platforms have been the most significant work by the scientists around the world since the beginning of 2020.

**Main body of the abstract:**

While positive results are being noticed with the currently used vaccines, the emerging variants of SARS-CoV-2 as well as the second wave of COVID-19 pandemic put the global public health in the deadliest health issue. Present review attempted to focus on the development of the current COVID-19 situation in the light of knowledge gathered from the recently published literature. An important facet regarding the COVID-19 severity is the avoidance of host immunity by the SARS-CoV-2 and its variants. Indeed, the genetic similarities between SARS-CoV-2, SARS-CoV-1 and the Middle East respiratory syndrome coronavirus (MERS-CoV) showed the viral escape strategies of the protective host immunity which appeared as the major problem for the effective vaccine development.

**Short conclusion:**

Present review discussed the prescribed platforms of vaccine development and pondered on the cellular and humoral immune responses by vaccines; and apart from vaccination approaches, non-pharmaceutical intervention approaches have also been pondered based on modeling rules.

## Background

The speed of the 2019–2020 severe acute respiratory syndrome coronavirus-2 (SARS-CoV-2) transmission as well as its rate of increase in numbers is much faster (as the COVID-19 pandemic has already been spread over all around the world causing 5,518,343 deaths out of 318,648,834 infected cases (as of January 15, 2022) compared to those of the other coronaviruses SARS-CoV (2003) and the Middle East respiratory syndrome coronavirus (MERS-CoV in 2012) (World Health Organization 2022). Both SARS-CoV and MERS-CoV originated from bats and then SARS-CoV particles were transmitted to humans from market civets; while MERS-CoV from dromedary camels; and in case of SARS-CoV-2, the origin was bat and the circulation has been facilitated by human-to-human transmission via the respiratory droplets (Noor 2021a). The mild symptoms due to SARS-CoV-2 are the onset of fever, dry cough and fatigue leading to dyspnoea, myalgia, etc., and in severe cases, the acute respiratory distress syndrome (ARDS) develops (Noor and Maniha 2020; Korber 2020). Lots of reports are available regarding the details of coronavirus genome, virulence, pathogenesis, host immune responses, viral mutations have been elucidated very clearly along with the possible target sites (Noor 2021a, 2020; Noor and Maniha 2020; Korber 2020).

Indeed, to mitigate the COVID-19 disease extensive knowledge-based and technical efforts are being given by scientists around the world emphasizing on the establishment of potential anti-viral drugs; and already the emergency medicine remedisivir has been endorsed together with other drugs like chloroquine/hydroxychloroquine, ribavirin, favipiravir, cepharanthine, opinavir/ritonavir, arbidol, etc. (Noor 2020). Besides designing anti-viral drugs, vaccination against COVID-19 have been considered as an effective prophylactic strategy for the control and prevention of the pandemic since the time COVID-19 started (Noor 2021a, 2020; Ura et al. 2021). In view of the huge acceleration of current COVID-19 fatality (especially with the commencement of the second wave), the mutation dynamics of SARS-CoV-2 is under analysis by the bioinformatics methods in order to infer the viral spreading mechanism; and a hotspot mutation was initially identified at position of D614 (resulting in the G614 variant, causing the replacement of glycine) within the receptor binding domain (RBD) of the viral spike (S) protein which accelerates the transmissibility of the mutant (Korber 2020; Ura et al. 2021). Such a finding is of significance for vaccine development as this evokes vaccine-induced immunity targeting the RBD of the spike protein (Ura et al. 2021).

**Fig. 1:**
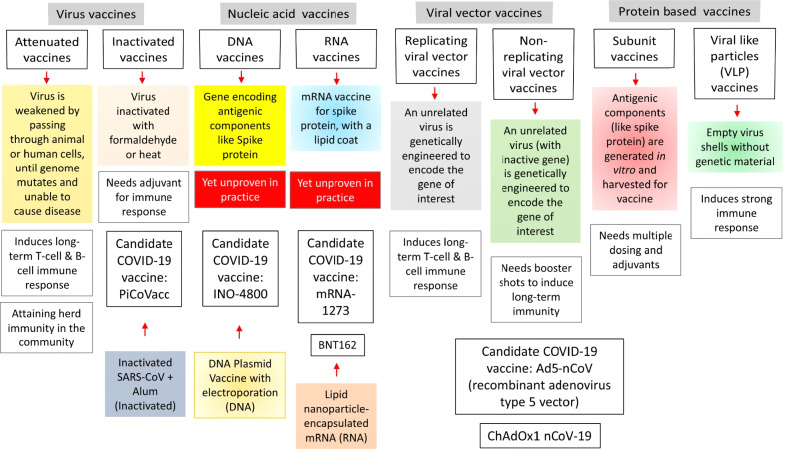
Vaccination strategies against COVID-19. The notable approaches for the development of candidate vaccines includes an array of technology platforms including live attenuated virus and inactivated virus approaches, the DNA- and RNA vaccines, use of virus-like particle (VLP), protein-based vaccines, and recombinant vaccines containing replicating- or non-replicating viral vectors. Phases of vaccine production have also been shown with a simple model

The development of a vaccine with a desirable long-lasting immunity against the viral infection is primarily based on the detection of synthetic/ natural antigen ensures that the vaccine dosage is safe and immunogenic as evident through the cell culture and animal study followed by the clinical trial authorization with the first phase (Phase I) of testing the vaccine safety, immune response, and the possible side effects involving limited numbers (20–100) of healthy volunteers (Ura et al. 2021; Noor 2021b). The second phase (Phase II) involves the dose response, schedule, assessment of vaccine safety, host immune response and the method of vaccine delivery involving diverge group of hundreds of volunteers (Noor 2021b; Mercado et al. 2020). Phase III involves thousands of target population with the testing for vaccine efficacy and safety with a fruitful outcome for biological license; and phase IV includes post marketing surveillance (Noor 2021b). The stages of vaccine development along with the manufacturing process, the categories of vaccines based on different platforms of vaccine design, vaccine immunology; and the candidate COVID-19 vaccines have been well discussed by Khuroo and colleagues at the beginning of the pandemic, as shown in Fig. 1 (Noor 2021b). However, by this date a bunch of commercial COVID-19 vaccines manufactured in different platforms already in use commercially of which ChAdOx1 nCoV19/AZD1222 (Oxford/AstraZeneca), BNT162b2 vaccine (Pfizer-BioNTech) and mRNA-1273 vaccine (Moderna), Gam-COVID-Vac-Lyo/Sputnik (Gamaleya), BB152/Covaxin (Bharat Biotech), and CoronaVac (SinoVac) vaccines are noteworthy (Ura et al. 2021; Noor 2021b, 2021c; Mercado et al. 2020; Diaz and Vergara 2021; Chung et al. 2021). These vaccines have been found to trigger cellular immunity including the elicitation of the antigen specific T cells, and provoking the T cell cytotoxicity; and also provoking the humoral immune response through the production of IgG (Noor 2021c).

However, the dawning of the second wave of COVID-19 as well as the emerging new variants of the SARS-CoV-2 raised the question about the efficacy of these vaccines since this new wave are largely affecting the younger individuals without posing much symptoms while the older persons are apparently being seemed to be less affected (Ura et al. 2021; Diaz and Vergara 2021; Chung et al. 2021). Surprisingly the typical mild symptoms are remaining absent in this wave rather resulting a sudden problem in the oxygen saturation levels and severe lung injury (Noor 2021a). Such a situation is currently being handled by the preventive measures like self-isolation or governmental lockdown; however, a sound approach in medication is essential for the long-lasting mitigation of COVID-19 (Noor 2021a, 2020, 2021b; Noor and Maniha 2020; Korber 2020; Ura et al. 2021). Therefore, extensive research and increased number of clinical trials with possible variations in the current vaccine composition is essential. Furthermore, possible improvements in the vaccine development platforms based on emerging variants of SARS-CoV-2 is necessary in the current fatal COVID-19 situation. Traditionally, vaccine development includes the range of technology platforms being evaluated, including live attenuated virus and inactivated virus approaches, the DNA vaccine or RNA vaccine, virus-like particle (VLP), protein-based vaccines, vaccines consisting of viral vectors (both replicating and non-replicating), etc. (Fig. 1). Another important consideration for vaccine development underlies the accuracy of the stimulation of active humoral and cellular immune response without the adverse effects or enhanced disease upon vaccination. Lots of reports have been published based on such information with a fine-tuned approach.

Along with the implementation of antiviral drugs and effective vaccination strategies to mitigate COVID-19, an important issue has to be pondered that many places around the world may lack the COVID-19 drugs and vaccines; thereby requiring lockdown and quarantine/ isolation concept (Sarkar et al. 2020). Interestingly, as reported by Sarkar et al. (2020) (whose team worked on mathematical modeling rules for the COVID-19 cases from the day of its commencement, and simulated all the required parameters for 260 days for all the states of India in order to study the SARS-CoV-2 dynamics), the basic reproduction number of SARS-CoV-2 was found to be reduced (in a mathematical modeling rules) as a result of the reduced contact rate (achieved through quarantining the susceptible individuals, restrictive social distancing, restricting travelling, and contact tracing) between the uninfected healthy individuals and the SARS-CoV-2 infected individuals (Sarkar et al. 2020).

Present review focused on the vaccine induced adaptive immunity, vaccine safety, and the prominent candidate vaccines principally categorized within inactivated vaccine, RNA- and DNA vaccine, and recombinant vaccines. Although the mode of actions and potential target of actions for the vaccines have been well discussed in the previous reports, the present review emphasized on the updated information for the sake of better knowledge in the current dreadful situation. Such knowledge would be beneficial for understanding (1) the actual strategies to combat the new variant strains of SARS-CoV-2 with the vaccine formulations triggering the long-lasting immunity within hosts; (2) development of successful vaccines with adequate clinical trials concerning the efficacy and safety of the vaccine(s). Finally, the need for preventive care in such a fatal condition of the COVID-19 pandemic has been clearly emphasized in the current review.

## Main text

### How would the vaccines elicit the adaptive immunity against COVID-19?

During the vaccine development, the major difficulties arise through the selection of appropriate vaccine platform, the uncertainty regarding the elicitation of sustainable, long-lasting immunity, and most importantly, to manage the uncontrolled cytokine storm which is a signature immune response upon the entry of SARS-CoV-2 particles either naturally or artificially (Noor 2021a, 2020, 2021c; Ura et al. 2021; Diaz and Vergara 2021; Chung et al. 2021). Another important aspect is to develop a safe and effective vaccine with the required the antigenicity and immunogenicity (Ura et al. 2021; Noor 2021c). The RBD of SARS-CoV-2 spike (S) protein has been shown as the most effective vaccine target as the RBD binds to the angiotensin converting enzyme-2 (ACE-2) receptor of the host during the viral entry (Noor 2021a, 2021c; Ura et al. 2021). However, a problem currently is being encountered regarding the mutation within the spike (S) protein since many vaccines are the so called “S-only” (full S protein or the S1/S2 subunit) vaccines (Chung et al. 2021). Hence, vaccines should be developed in a way that these can be targeted to not just the S protein but also to the membrane (M) protein, envelope (E) protein, nucleocapsid (N) phosphoproteins and the hemagglutinin-esterase (HE) protein of the virus. This is to be recalled that the S protein (the major target for vaccination) of SARS-CoV-2 which consists of S1, RBD and S2 subunits (containing the fusion peptide and two heptad repeats required for the viral fusion into the host membrane), binds to the ACE2 receptor (expressed in the nasal epithelium, lung, heart, kidney, and intestine) on the host surface with the aid of RBD, followed by the cleavage of S protein by the transmembrane protease serine (TMPRSS2) into S1 and S2 subunits during the viral entry into the host via membrane fusion (Noor 2021a, 2020, 2021c; Chung et al. 2021). For appropriate vaccine designing, such mechanism of SARS-CoV-2 entry within the host has to be always considered.

However, upon recognition of SARS-CoV-2 by the lung epithelial cells (binding to epithelial cells via ACE2 receptors), innate and adaptive immunity is triggered within the host (Noor 2021a). The toll like receptors (TLRs) 3, 7/8 and the retinoic acid-inducible gene I (RIG-I)/ melanoma differentiation associated gene-5 (MDA-5) act as intracellular signals for the production of cytokines, activation of transcription factors, interferon regulatory factor (IRF) 3/7 and the nuclear factor kappa light chain enhancer of activated B cells (NF-kB) signal transduction pathways, and for the production of type I interferons (IFNs) which are supposed to create an anti-viral state by producing inflammatory cytokines and chemokines as well as anti-viral enzymes (Noor 2021a, 2020; Chung et al. 2021). Alveolar macrophages carrying an array of cytokines rush towards the site of infection; and the resident dendritic cells (DCs) facilitate the trapping of viral antigen which is processed for presentation by the major histocompatibility complex (MHC) I to CD8^+^ T cytotoxic cells (Tc) and MHC II to CD4^+^ T cells; and afterward the CD4^+^ T helper cells (Th) stimulate B cells (triggering antibody-mediated immune response), the CD8^+^ T cells (mediating cellular immune responses); and help in the development of memory cells (Noor 2021a, 2021c; Chung et al. 2021). Th1 cells secrete the granulocyte–macrophage colony-stimulating factor (GM-CSF) which in turn activates the CD14^+^ CD16^+^ monocytes to elicit interleukin (IL)-6 whereas Th17 cells are involved in the production of IL-17 to further recruit phagocytes/ antigen processing cells (APCs) and stimulate the production of IL-1, IL-1β, IL-6, etc. (Noor 2021a; Chung et al. 2021).

Usually, the antigens (S protein, S1 or S2 subunit, RBD, nucleoprotein or the multiple epitopes) within the vaccines mount the specific immune response without resulting in disease (Noor 2021a; Diaz and Vergara 2021; Chung et al. 2021). Upon the SARS-CoV-2 attack, memory cells can be activated and differentiated into other cells types; i.e., the effector T cells and the antibody-producing B cells which are supposed to neutralize the virus (Noor 2021c; Diaz and Vergara 2021). The virus coated with the neutralizing antibodies cannot interact with angiotensin converting enzyme-2 (ACE-2) receptor of the host with its RBD site of the S protein (Noor 2021c). Antigenic responses based on T cells depend on the recognition and killing of infected cells (Chung et al. 2021). The antigens are processed by the DCs, displaying the small antigenic peptides at the cell surface of the MHC class I and class II molecules. MHC class I-peptide complexes are subsequently recognized by cytotoxic T cells (CD8^+^), differentiating into cytotoxic effector cells (Tc) killing the infected cells or the viruses directly; whereas the helper T cells (Th) (CD4^+^) recognize MHC class II-peptide complexes and differentiate in the effector cells generating Th1 (augmenting the CD8^+^ T-cell differentiation) and Th2 which further activate B cells to provoke the plasma cells to generate antibody production, and may produce cytokines which inhibit CD8^+^ T-cell differentiation (Noor 2021a, 2021b, 2021c).

**Fig. 2:**
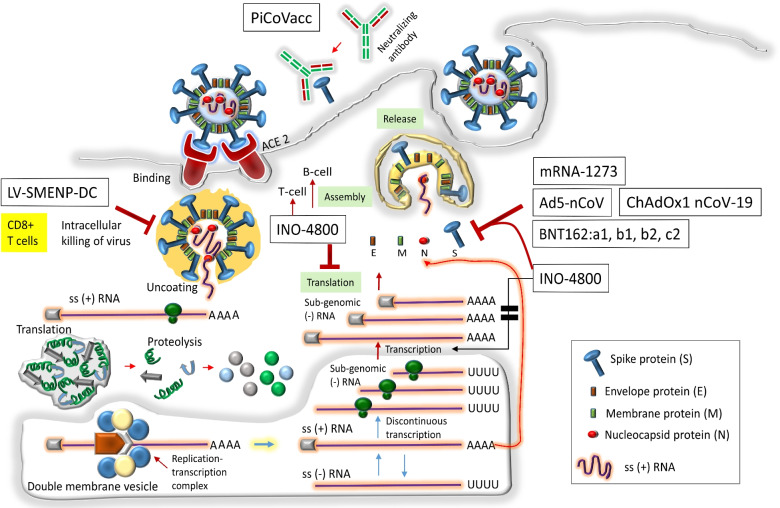
The SARS-CoV-2 life cycle has been shown whereby the possible targets for the candidate vaccines are pointed as well. Details are described in the text

As shown in Fig. 2, one of the candidate vaccines PiCoVacc facilitated in animal models to increase titer of the neutralizing antibodies which could bind to the spike (S) protein. The target sites for the candidate vaccines mRNA-1273 (messenger RNA vaccine), recombinant adenovirus type-5 vectored COVID-19 (Ad5-nCoV) vaccine, INO-4800 (DNA vaccine), BNT162: a1, b1, b2, c2 (RNA vaccine), ChAdOx1 nCoV-19 (non-replicating viral vector), LV-SMENP-DC (recombinant vaccine), and pathogen-specific aAPC (recombinant vaccine) are the S protein of the SARS-CoV-2. The viral vector vaccine, ChAdOx1 nCoV-19 (Oxford/ AstraZeneca) encodes and stabilizes S protein; and evokes S protein specific responsiveness of CD4 + T cells, CD8 + cells and the T helper cell 1 (Th1) responses. The IgG anti–spike (S) protein response as well as the secondary T-cell responses have been noticed by the NVX-CoV2373 (Novavax) nanoparticle vaccine; and BBIBP-CorV (Sinopharm) vaccine has been found to elicit the production of neutralizing antibodies. The Pfizer-BioNTech (BNT162b1) and Moderna (mRNA 1273) vaccines are currently under phase III trial in which mRNA that encodes the spike protein is shielded with a submersible lipid nanoparticle; and upon its administration into the host, spike protein is expressed which in turn provokes the host cellular and humoral immunity (Noor 2021a).

### Sustainability of adaptive immunity and protection against re-infection

As the COVID-19 candidate vaccines and natural SARS-CoV-2 infections are supposed to have analogous immunological responses within host, the clear understanding and deciphering the host immune responsiveness against viral infection goes a long way to the possible durability of adaptive immunity upon vaccination (Noor 2021a, 2021c; Diaz and Vergara 2021). Upon the SARS-CoV-2 infection, both IgM and IgG antibodies appear at the 10th day against internal nucleoprotein (N) and mostly against the spike (S) protein (Chen and Li 2020). Interestingly, the mutations in SARS-CoV-2 have been noticed as very slow and mild resulting in unchanged sequences in the mutants as in the parent strain so that a stringent set of B cell and T cell epitopes derived from the S- and N- proteins can be used experimentally for the development of vaccines against SARS-CoV-2 (Noor 2021b, 2021c; Dorp et al. 2020). It is to be noted that the duration of immunity against SARS-CoV-1 and MERS-CoV was long-lasting with the appropriate induction of T cell and B cell immune response (Noor 2021b; Zheng et al. 2021). A retrospective cohort study using 404 serum specimens from 172 convalescent COVID-19 patients (all of them initially contained the anti-SARS-CoV-2 IgM/IgG at the onset of COVID-19 symptoms) in Wuhan during the epidemic peak revealed that the antibody titer (S1-total antibody and RBD-IgG levels) during the convalescence were high among 98% of the population up to 6 months minimum thereby projecting an optimistic motion for the anti-secondary infection (Zheng et al. 2021). Such human studies are also consistent with the results of the animal study whereby two monkeys recovering from COVID-19 were found to be protected from re-infection by the SARS-CoV-2 (Noor 2021b). A candidate vaccine PiCoVacc Sinovac Biotech, administered to mice, rats, and non-human primates (three immunizations of 3 μg or 6 μg per dose) revealed that the raised antibodies had neutralizing capacity against SARS-CoV-2 strains (Noor 2021b; Gao et al. 2020). This vaccine facilitated in the animal models to increase the titer of the neutralizing antibodies which could bind to the spike (S) protein (Noor 2021b; Gao et al. 2020). Therefore, it can be deduced that the vaccination strategies may possess the ability to prevent re-infection.

However, it is to be noted that the first experimental SARS-CoV vaccines (inactivated viruses or virus vectors as shown in Fig. 1) were found to elicit tissue infiltration of lymphocytes, monocytes and eosinophils in experimental animals (Noor 2021a, 2021c; Tseng et al. 2012). A detailed analysis suggested that T_H_17 responses (Th17 cells playing a role in host defense against the viruses by mediating the recruitment of neutrophils and macrophages to infected tissues) might be responsible for such cellular responses (Noor 2021c). Such an observation is suggestive of the association between T_H_17 cell responses and the production of IL-6 and IL-8, the major pro-inflammatory cytokines strongly elevated in the COVID-19 patients (Noor 2021c). Besides, IL-17 might promote the activation and recruitment of eosinophils from bone marrow and the subsequent deposition into target organs like lungs (Noor 2021c; Hotez et al. 2020). Adjuvants are known to enhance immunogenicity as well as enables vaccination protection confirmation. Use of aluminum-containing adjuvant was noticed to promote the T_H_2-type immunity (involved in reducing the asthma) which in turn decreased the extent of inflammation (Tseng et al. 2012).

### Vaccines protecting from infection or causing the disease enhancement?

Vaccine development requires manufacturing platforms, structure-based antigen design, knowledge on computational biology to predict target sites, bioinformatics analysis with protein engineering, and finally sufficient trials both in cell culture/animal model and the human clinical trials. However, the most important concern during designing a vaccine is to extrapolate whether the vaccine really protects the host from the infection or it rather results in the escalation of the current infection state, leading to the extreme severity or death. For example, antibodies that bind SARS-CoV-2 spike (S) protein without neutralizing capacity certainly may trigger COVID-19 through elevated viral replication or by formation of immune complexes leading to lung inflammation. As reported earlier, more than 200 vaccine candidates are in pre-clinical trials of which more than 10 are in phase II/III trials (Noor 2021a; Smatti et al. 2018; Zellweger et al. 2020). While maximum of these vaccines is showing the effectiveness, even though the possibility of the vaccine-induced disease development upon vaccination has already been flagged as a conceivable safety concern requiring specific concentration by the international regulatory bodies (Zellweger et al. 2020). It is to be noted that no vaccination is completely free of complications and undesirable side effects (Noor 2021a). For example, (1) the ChAdOx1 nCoV-19 (NCT04324606) vaccine have been noticed with fever, pain, muscle aches, chills and headache; (2) previously the recombinant vaccinia virus Ankara expressing the S protein of SARS-CoV-1 was found to increase hepatitis in ferrets (Haidere et al. 2021). Therefore, the ongoing vaccine manufacturers need to resolve the issue of vaccine safety and effectiveness.

Actually, masking of the non-neutralizing epitopes of spike (S) protein by glycosylation or by selecting critical neutralizing epitopes of the S antigen may stimulate a stouter protective immunity against SARS-CoV-2 infection (Noor 2021b). Another immunological point to ponder is that while developing vaccines against SARS-CoV-1 and MERS-CoV, antibody-dependent enhancement (ADE) was markedly noticed, causing the response towards the viral infection more symptomatic, and thereby rendering the ongoing second wave of COVID-19 infections more dreadful as evident from a number reinfection cases as well as getting the COVID-19 patients quickly to the mortality (Noor 2021a, 2021c). There is another vaccine associated complication named as vaccine-associated enhanced respiratory disease (VAERD), which occurred in young children in the 1960s upon the administration of the whole-inactivated virus vaccines for measles and respiratory syncytial virus (RSV) (Munoz et al. 2021). However, the vaccine formulation and delivery can also be fashioned to influence the T cell functions along with the response patterns. It is to be noted that the gene -based delivery is likely to induce CD8^+^ T cells and generally drive a CD4^+^ T helper 1 cell (T_H_1)–type immune response, which is obviously possess sound anti-viral traits (Noor 2021c; Munoz et al. 2021).

### Implication of the candidate vaccines

The vaccine development landscape for COVID-19 is almost dynamic, with a range of divergent technological platforms and innovations by different research teams around the world. The different vaccination strategies, approaches and efficacy are being tested rapidly with the expectation for selecting an appropriate vaccine candidate. Platform technology is an effective strategy for developing candidate vaccines, posing automation, speed of manufacture, scope of development of several prototype vaccines from the single system, etc. (Noor 2021b, 2021c; Thanh et al. 2020; Kaur and Gupta 2020; Mukherjee 2020; Li et al. 2021). The novel platforms based on DNA or mRNA revels the great suppleness regarding antigen manipulation. mRNA-based vaccines may also be developed by platform technology with the accuracy of target and delivery as predicted (Noor 2021b). The most advanced COVID-19 vaccine candidates have so far been listed in Table 1. As of July 2020, 158 vaccine candidates (based on various platforms including the inactivated or live attenuated viruses, protein sub-unit, VLP, viral vector, DNA, RNA, and nanoparticles) appeared of which 135 underwent clinical trials (Kaur and Gupta 2020). Currently, 23 vaccines already have gone under clinical evaluation while at least 140 candidate vaccines are in the preclinical evaluation (Li et al. 2021). As stated earlier, already several vaccines have been launched in the global market for COVID-19 mitigation of which Pfizer-BioNTech (BNT162b1) and Moderna (mRNA 1273) vaccines, ChAdOx1 nCoV-19 (Oxford/ AstraZeneca) vaccine, JNJ-78436735/Ad26.COV2.S (Johnson&Johnson) vaccine, NVX-CoV2373 (Novavax) vaccine, BBIBP-CorV (Sinopharm) vaccines have shown positive outcome (Ura et al. 2021; Noor 2021b, 2021c). Nevertheless, the escaping strategy of the host immunity by the emerging SARS-CoV-2 variants needs to be carefully address by more clinical trials and extensive research on vaccine composition since mutations in the viral spike (S) protein have made the virus incrementally lethal to mass public (Korber 2020; Noor 2021c; Diaz and Vergara 2021; Chung et al. 2021; Chen and Li 2020; Dorp et al. 2020; Zheng et al. 2021). Because of the incidences of evolvement of the spike protein variants, continuous surveillance of SARS-CoV-2 genome and the efficiency of implemented vaccines are of global health significance at the present time.

**Table 1:** Major vaccines in commercial use for COVID-19 mitigation together with clinical trials

Candidate vaccines	Vaccine composition	Current stage	Developer	Mode of action	References
PiCoVacc (inactivated vaccine)	Inactivated SARS-CoV particles + Alum adjuvant (Inactivated)	Phase III	Sinovac Biotech	Produces neutralizing antibodies against spike (S) protein	Ura et al. (2021), Noor (2021b), Gao et al. (2020)
BNT162: a1, b1, b2, c2 (RNA vaccine)	Lipid nano-particle (LNP)-encapsulated mRNA vaccine encoding spike (S) protein	Phase III	BioNTech, Fosun Pharma and Pfizer	Possible target: spike (S) protein	Ura et al. (2021), Noor (2021b), Noor (2021c)
ChAdOx1 nCoV-19/AZD1222 (Non-replicating vector vaccine)	Weakened version of a common cold virus (adenovirus): the genetic material has been added to the ChAdOx1 construct	Phase III	AstraZeneca, University of Oxford	Possible target: spike (S) protein	Ura et al. (2021), Noor (2021c)
mRNA-1273 (RNA vaccine)	Lipid nano-particle (LNP)-encapsulated mRNA (RNA) vaccine encoding spike (S) protein	Phase III	Moderna, NIAID	Possible target: Spike (S) protein	Ura et al. (2021), Noor (2021c)
Ad5-nCoV (Recombinant vaccine)	Adenovirus type 5 vector; i.e., non-replicating viral vector (expressing S protein)	Phase III	CanSino biologicals	Possible target: spike (S) protein	Ura et al. (2021), Noor (2021c)
INO-4800 (DNA vaccine)	DNA plasmid encoding S protein delivered by electroporation	Phase I-II	Inovio pharmaceuticals, CEPI, Korean Institute of Health, International Vaccine Institute	Possible target: spike (S) protein; triggers the T cell- and antibody response; hinders viral mRNA transcription	Ura et al. (2021), Noor (2021b), Kaur and Gupta (2020), Li et al. (2021), Baden et al. (2021)
Ad26.COV2.S (Ad26 vector based vaccine)	Seven variants of the SARS-CoV-2 spike (S) protein sequences which have been codon optimized and synthesized	Phase III	Janssen Vaccines and Prevention B.V. (Johnson & Johnson)	The most divergent group of vaccine with S protein variants as target; triggers (1) S-specific and RBD-specific antibody responses; (2) cellular immune responses: IFN-γ + CD4 + and CD8 + T cell responses	Ura et al. (2021), Mercado et al. (2020)
Gam-COVID-Vac/ Sputnik V COVID-19 vaccine (two-vector vaccine)	Recombinant replication-defective adenovirus serotype 26 + serotype 5	Phase III	Gameleya Research Institute of Epidemiology and Microbiology	Possible target: spike (S) protein	Ura et al. (2021)
LV-SMENP-DC vaccine (lentiviral vector vaccine)	Prepared by engineering dendritic cells (DC) with the lentiviral vector expressing synthetic minigene which are based on domains of selected viral proteins	Phase I	Shenzhen Geno-Immune Medical Institute	Possible target: structural proteins of SARS-CoV-2; activates the cytotoxic T cells	Noor (2021b)

Vaccine safety and immune response have been determined through the randomized double-blinded placebo-controlled mechanism

Among the commercial vaccines in current date, two COVID-19 mRNA vaccines; i.e., BNT162b2 mRNA Covid-19 vaccine and mRNA-1273 SARS-CoV-2 vaccine have been found to be effective (approximately 95%) from Phase III clinical trials as well as from the respondents’ survey (Noor 2021c; Polack et al. 2020; Baden et al. 2021; Heinz and Stiasny 2021). Both of them consist of a nucleoside-modified mRNA which encodes the full-length sequence of S protein added with two stabilizing proline mutations within S2; and their delivery mode employs lipid nanoparticles for delivery (Noor 2021c; Kaur and Gupta 2020; Mukherjee 2020). The adenovector COVID-19 vaccines especially, the ChAdOx1-S/AZD1222 vaccine manufactured by the University of Oxford/AstraZeneca (AstraZeneca, Cambridge, UK), and the Sputnik V vaccine manufactured by the Gamaleya Institute in Moscow, Russia met the demand for the prophylactic mass immunization against the COVID-19 pandemic (Heinz and Stiasny 2021; Coughlan 2020). Usually, high doses of vector particles are administered which are subsequently recognized by the host immune sensors to induce the release of cytokines and chemokines that unfortunately account for vaccine associated side effects (Coughlan 2020). As stated elsewhere, whereas the adenovector vaccines express full-length S protein; however, the Janssen hAd26 vaccine (termed Ad26.COV2.S) consists of steadying mutations rendering this vaccine most divergent; and is currently being evaluated in clinical trials. (Mercado et al. 2020; Coughlan 2020). The single shot of an Ad26 vector encoding the prefusion stabilized S immunogen (S.PP, containing the wild type leader sequence, the full-length membrane-bound S, mutation of the furin cleavage site, and two proline stabilizing mutation) elicited the production of the neutralizing antibodies which imparted a complete protection against the viral challenge in 5 of 6 rhesus macaques (Mercado et al. 2020). Vaccines manufactured Sinovac Biotech (Sinovac, Beijing, China) and Sinopharm (Sinopharm, Beijing, China) are the conventional inactivated whole-virus vaccines which have 86% record of vaccine efficiency (Heinz and Stiasny 2021; Forni and Mantovani 2021). It is to be noted that Bharat Biotech in India also manufactures a similar type of vaccine as well as the same is produced by the French company Valneva whereby the SARS-CoV-2 is grown in Vero cells followed by chemical inactivation by β-propiolactone (BPL), purification, and addition of the required adjuvants (Heinz and Stiasny 2021). Among the subunit COVID-19 candidate vaccines, the protein subunit vaccines have been quantified up 32% which is so far the highest in number out of 63 candidates currently in clinical progress (Heinz and Stiasny 2021). One of such vaccines namely NVX-CoV2373 (Novavax, Gaithersburg, MD, USA) has completed Phase III clinical trials (whereby the antigen was a recombinant full-length S protein with stabilizing mutations) with an outcome of approximately 90% success in a trial in the UK (Wadman and Cohen 2021). However, the rate of success was hardly 60% in South Africa (Heinz and Stiasny 2021).

### Vaccines against SARS-CoV-2 variants: what is the current status?

The evaluation of the effectiveness of a vaccine is actually based on the Phase III randomized controlled trials (RCTs) which relate the frequency of COVID-19 in large groups of vaccinated and non-vaccinated individuals (Noor 2021c; Forni and Mantovani 2021; Wadman and Cohen 2021). Such evaluation detects whether one, several or none of the new COVID-19 vaccines is/are capable to protect infection with the utmost effectiveness or only marginally; and also, whether there are any side effects or not (Forni and Mantovani 2021). As stated earlier, the prime concern regarding the mitigation of the COVID-19 pandemic in the current situation of the second wave is to carefully analyze the impact of the mutations of the spike proteins generating new variants of the virus: the UK variant B.1.1.7, i.e., N501Y.V1, known as VOC-202012/01; the South African Variant B.1.351, 20H; i.e., N501Y.V2 (501Y.V2); and the Brazilian Variant B.1.1.28.1; i.e., P.1 (501Y.V3, 20 J) (Noor 2021a). Indeed, along with the increased transmissibility of SARS-CoV-2, several mutations have been noticed at several important antigenic sites (for example, mutations in the S protein) of SARS-CoV-2 (Noor 2021a; Korber 2020; Diaz and Vergara 2021; Heinz and Stiasny 2021). However, the N501Y.V1 was neutralized the BioNTech/Pfizer mRNA and the Moderna mRNA vaccines; N501Y.V2 strains were found to be neutralized by the Oxford/Astra Zeneca vaccine (Heinz and Stiasny 2021). Another important point is to note that the recent reports of lower efficacy rates of Novavax subunit vaccine (Phase III clinical trials) in South Africa than that in the UK and Janssen Adeno26 vaccine in Latin America and South Africa than that in the USA are of significance since such fluctuation in vaccine efficiency based on the geographical locations may not accurately mitigate the present SARS-CoV-2 variants revealing an issue of variant-vaccine combination (Heinz and Stiasny 2021). Different vaccines have different efficacy and variants like beta (highly resistant) can influence or reduce the efficacy. Thus, intensive surveillance of the emerging variants based on their genome sequencing and bioinformatics analysis, the transmission dynamics, study of the increasing risk of dodging host protective immunity by new strains urge the massive improvement in the current vaccine research (Korber 2020; Ura et al. 2021; Noor 2021c; Diaz and Vergara 2021; Heinz and Stiasny 2021).

### Measures to alleviate COVID-19 pandemic by modeling rules: beyond the effort to develop the vaccines

Considering the above discussion about the coronavirus transmission speed, the second wave commencement, and finally, the questionable efficiency of some commercialized vaccines, the world has undergone an uncertainty due to the COVID-19 pandemic. Although COVID-19 related data from different databases are accessible in an orchestrated manner (i.e., the genome sequences of the SARS-CoV-2 variants in different geographical locations as well as the immune responses against different vaccines); yet queries still remain unsettled to precisely forecast the transmission dynamics of SARS-CoV-2 in any area within the world. As stated in Introduction section, another important issue is that many locations are still devoid of repurposed drugs let alone COVID-19 vaccines. Therefore, preventive measures like implementation of self-isolation, public lockdown, quarantine against COVID-19 are essential; and the uncertainty of the authentic data related to COVID-19 relating to the accurate number of infected individuals which in turn may direct to the ambiguous outcomes and wrong predictions must be resolved by some modeling rules (Sarkar et al. 2020; Battegay et al. 2020; Kretzschmar and Wallinga xxxx).

The model proposed by Sarkar et al., 2020, actually scrutinized the dynamics of six parts: susceptible (S), asymptomatic (A), recovered (R), infected (I), isolated infected (*I*_*q*_) and quarantined susceptible (*S*_*q*_) which were collectively expressed as *SARII*_*q*_*S*_*q*_; on which a sensitivity analysis was conducted to detect the strength of such mathematical model predictions according to the values of the parameters as assessed from the tangible data on the ongoing COVID-19 wave (Sarkar et al. 2020). More descriptively, the model parameters set by Sarker et al. (2020) have been assessed through initial population size fitted to the model simulation with the observable COVID-19 cases with the probability of disease transmission (*β*_*s*_), quarantined rate of susceptible individuals (*ρ*_*s*_), contact rate of entire individuals (ϵ_*s*_), probability rate at which the asymptomatic individuals may develop the clinical symptoms (*γ*_*a*_), recovery rate of the asymptomatic SARS-CoV-2 infected individuals (*ξ*_*a*_) and the rate of recovery for COVID-19 patients (*ξ*_*i*_) on the basis of the daily new COVID-19 cases (Sarkar et al. 2020). The need for such modeling rules came out from the fact that in India some places lack specific pharmaceutical interventions for which some essential measures like social distancing, closing the educational organizations (schools/ colleges/ universities), shutting down all the offices, restricting all the public gathering places like churches, bars, theatres, restaurants, and all other social places of mass gathering were taken by authorizing strict quarantine, surveillance of the possible transmission, contact tracing, etc. (Battegay et al. 2020). Indeed, such predictive mathematical models go a long way to infer the progression of a pandemic as well as to design effective approaches to halt contagious diseases in case of the lack of any specific antiviral drugs and effective vaccine(s) (Sarkar et al. 2020; Battegay et al. 2020; Kretzschmar and Wallinga xxxx). Around 100 years ago, a series of reports appeared whereby the dynamics of disease transmission was given in terms of a system of differential equations; known as the concept of a threshold quantity separating different dynamic regimes. If the basic reproduction number is found above that threshold value confers that a disease is infectious among a susceptible population (Kretzschmar and Wallinga 2009). Such an estimation is quite interesting in context of vaccination since it leads to the perception of herd immunity, a state whereby vaccination of the entire population is not at all essential to mitigate the infectious disease (Kretzschmar and Wallinga 2009). Such a state of herd immunity can be achieved by appropriate quarantine and isolation approaches where the effective vaccination is not possible. However, this is to be recalled that in context of current COVID-19 pandemic, Sarkar et al., 2020 described the quarantine concept as the separation of SARS-CoV-2 infected populations from the susceptible individuals so that further progression of the disease cannot take place as evident by the clinical symptoms; and the isolation has been described as the detachment of the COVID-19 patient from the healthy individuals (Sarkar et al. 2020).

Regarding such quarantine and isolation aspects, India may be the best example as this country implemented these social distancing approaches with the modeling rules when the pandemic had been first confirmed in Kerala on January 30, 2020 after a student came back from Wuhan (Samui et al. 2020). The declaration of 14 h voluntary public curfew on March 22, 2020; a 21 days nationwide lockdown from March 25, 2020 to April 14, 2020; extending the lockdown up to May 03, 2020; creating public awareness through the internet and social media platforms by the health authorities/government officials by informing about the hospitalized patients, symptomatic individuals, and mandatory quarantine of the asymptomatic individuals were the effective steps taken by the Government of India to halt the SARS-CoV-2 transmission (Samui et al. 2020; Rai et al. 2021). The principles behind such measure was the mathematical modeling to understand how transmissible COVID-19 disease was that time; at which phase the infectivity gets high; how dreadful the disease was and what the symptoms were; and how much effective non-pharmaceutical interventions could be conducted (Battegay et al. 2020; Kretzschmar and Wallinga 2009; Samui et al. 2020; Rai et al. 2021). This is to be recalled that dynamic modelling rules included the total population N(t) which was further classified into six subpopulations (classes) named as: susceptible S(t), exposed E(t), asymptomatic A(t), clinically ill or symptomatic I(t), hospitalized H(t) and recovered R(t) individuals (Battegay et al. 2020; Khajanchi et al. 2021). Such modeling concluded the strict rules of avoidance of mass gatherings, maintaining the social distancing and implementation of extensive lock-down; providing personal safeguard to the frontline workers; in time dissemination of COVID-19 status (i.e., the number of symptomatic/ asymptomatic and hospitalized cases); and most importantly, accelerating public awareness on COVID-19 through media platforms (Khajanchi et al. 2021).

### Emerging variants of SARS-CoV-2 and the requirement for the non-pharmaceutical interventions

One thing is clear that compared to the infectivity and transmissibility posed by SARS-CoV-1 and MERS-CoV, the SARS-CoV-2 imparted high degree virulence and transmissibility, which in turn resulted in the rapid development of vaccines to mitigate the ongoing COVID-19 pandemic (Noor 2021a, 2021c; Ura et al. 2021; Diaz and Vergara 2021; Chung et al. 2021; Gao et al. 2020; Thanh et al. 2020). However, as stated earlier, besides the original Wuhan strain of SARS-CoV-2, the emerging variants of concern (VOC) and the variants of interest (VOI) brought about the reduced efficacy of the vaccines currently in use (Noor 2021b; Chakraborty et al. 2021; Ferré et al. 2021). After the initiation of the original SARS-CoV-2 strain, in late 2020 in the UK, several variants of concern like the alpha variant of B.1.1.7 lineage evolved in the late 2020 in the UK, the beta variant (B.1.351) developed in South Africa, the gamma variant of P.1 lineage emerged in Brazil, and the delta variant (B.1.617.2) emerged in India (Chakraborty et al. 2021). The current outbreak of the omicron variant of B.1.1.529 lineage initially originated in November 2021 in South Africa (Ferré et al. 2021). The variants of interest are the eta variant of B.1.525 lineage and the iota variant of B.1.526 lineage, both of which evolved in New York, USA, the zeta variant of P.2 lineage which emerged in Brazil; and the epsilon variant of B.1.427/B.1.429 lineage which was first detected in California, USA (Chakraborty et al. 2021; Ferré et al. 2021). Mutations in the S protein (i.e., amino acid changes; i.e., shifting, replacement, or deletion) of these strains are of major concern as the vaccines are mainly constructed to evoke the neutralizing antibodies against the S protein components (Chakraborty et al. 2021; Ferré et al. 2021). The mutated strains appear to be resistant against the vaccines which demand the booster dose of vaccines as well as the necessity of the non-pharmaceutical interventions as described above (Kretzschmar and Wallinga 2009; Samui et al. 2020; Rai et al. 2021; Chakraborty et al. 2021; Ferré et al. 2021).

## Conclusions

Understanding vaccine categorization and the implementation platform together with safe host immunological response is crucial for the determination of accuracy of the vaccines. The information provided in the current review regarding vaccine induced adaptive immunity as well as immunopathology, safe and protective function of a vaccine rather than accelerating disease severity; and about the potential candidate vaccines would be incremental to the existing knowledge on the COVID-19 vaccine research and development. Taking the Spike (s) protein mutations into account as well as on the basis of the current dreadful wave of COVID-19, a faultless platform of clinical trials reporting nonbiased data on the vaccine efficacy would be very helpful for the assessment of the vaccines which have been permitted for the current administration worldwide. Regular surveillance of the genome sequences of the spreading SARS-CoV-2 according to the geographic locations is necessary as well. Upon the observation of the unwanted inefficiency of vaccines to instigate cellular and humoral immunity within host, renovation of the current vaccines may be necessary; or even the necessity of construction and execution of the seasonal vaccines may come up for the better management of COVID-19 pandemic. Also, places where vaccination strategies are not implementable have been taken into account in the review; and a brief notion on modeling rules-based quarantine and isolation to mitigate the transmission of SARS-CoV-2 has been included which may help to create more public awareness during the current second wave of COVID-19 as well as in case of the reduction of vaccine effectiveness.

## Data Availability

Not applicable.

## Abbreviations

SARS-CoV-2: Severe acute respiratory coronavirus 2
COVID-19: Coronavirus disease 2019
MERS-CoV: Middle East respiratory syndrome coronavirus
ARDS: Acute respiratory distress syndrome
RBD: Receptor binding domain
VLP: Virus-like particle
ACE-2: Angiotensin converting enzyme-2
S: Spike
M: Membrane
E: Envelope
N: Nucleocapsid
HE: Hemagglutinin-esterase
TLRs: Toll like receptors
RIG-I: Retinoic acid-inducible gene I
MDA-5: Melanoma differentiation associated gene-5
IRF: Interferon regulatory factor
NF-kB: Nuclear factor kappa light chain enhancer of activated B cells
IFNs: Interferons
DCs: Dendritic cells
MHC: Major histocompatibility complex
Tc: T cytotoxic cells
Th: T helper cells
GM-CSF: Granulocyte–macrophage colony-stimulating factor
IL: Interleukin
APCs: Antigen processing cells
VAERD: Vaccine-associated enhanced respiratory disease
RSV: Respiratory syncytial virus
BPL: β-Propiolactone
RCTs: Randomized controlled trials
S: Susceptible
A: Asymptomatic
R: Recovered
I: Infected
*I*_*q*_: Isolated infected
*S*_*q*_: Quarantined susceptible
*β*_*s*_: Probability of disease transmission
*ρ*_*s*_: Quarantined rate of susceptible individuals
ϵ_*s*_: Contact rate of entire individuals
*γ*_*a*_: Probability rate at which the asymptomatic individuals may develop the clinical symptoms
*ξ*_*a*_: Recovery rate of the asymptomatic SARS-CoV-2 infected individuals
*ξ*_*i*_: Rate of recovery for COVID-19 patients
N(t): Total population
S(t): Susceptible population
E(t): Exposed population
A(t): Asymptomatic population
I(t): Clinically ill or symptomatic population
H(t): Hospitalized individuals
R(t): Recovered individuals

## References

[CR1] Baden LR, El Sahly HM, Essink B, Kotloff K, Frey S, Novak R (2021). Efficacy and safety of the mRNA-1273 SARS-CoV-2 vaccine. N Engl J Med.

[CR2] Battegay M, Kuehl R, Tschudin-Sutter S, Hirsch HH, Widmer AF, Neher RA (2020). 2019-novel Coronavirus (2019-nCoV): estimating the case fatality rate - a word of caution. Swiss Med Wkly.

[CR3] Chakraborty C, Sharma AR, Bhattacharya M, Agoramoorthy G, Lee SS (2021). Evolution, mode of transmission, and mutational landscape of newly emerging SARS-CoV-2 variants. mBio.

[CR4] Chen Y, Li L (2020). SARS-CoV-2: virus dynamics and host response. Lancet Infect Dis.

[CR5] Chung JY, Thone MN, Kwon YJ (2021). COVID-19 vaccines: the status and perspectives in delivery points of view. Adv Drug Deliv Rev.

[CR6] Coughlan L (2020). Factors which contribute to the immunogenicity of non-replicating adenoviral vectored vaccines. Front Immunol.

[CR7] Diaz RS, Vergara TRC (2021). The COVID-19 second wave: a perspective to be explored. Braz J Infect Dis.

[CR8] Ferré VM, Peiffer-Smadja N, Visseaux B, Descamps D, Ghosn J, Charpentier C (2021). Omicron SARS-CoV-2 variant: what we know and what we don't. Anaesth Crit Care Pain Med.

[CR9] Forni G, Mantovani A (2021). COVID-19 commission of Accademia Nazionale dei Lincei, Rome. COVID-19 vaccines: where we stand and challenges ahead. Cell Death Differ..

[CR10] Gao Q, Bao L, Mao H, Wang L, Xu K, Yang M (2020). Development of an inactivated vaccine candidate for SARS-CoV-2. Science.

[CR11] Haidere MF, Ratan ZA, Nowroz S, Zaman SB, Jung YJ, Hosseinzadeh H, Cho JY (2021). COVID-19 vaccine: critical questions with complicated answers. Biomol Ther (seoul).

[CR12] Heinz FX, Stiasny K (2021). Profiles of current COVID-19 vaccines. Wien Klin Wochenschr.

[CR13] Hotez PJ, Bottazzi ME, Corry DB (2020). The potential role of Th17 immune responses in coronavirus immunopathology and vaccine-induced immune enhancement. Microbes Infect.

[CR14] Kaur SP, Gupta V (2020). COVID-19 Vaccine: a comprehensive status report. Virus Res.

[CR15] Khajanchi S, Sarker K, Mondal J (2021) Dynamics of the COVID-19 pandemic in India. Quantitative Biology (Populations and Evolution). arXiv:2005.06286v2

[CR16] Korber B (2020). Tracking changes in SARS-CoV-2 spike: evidence that D614G increases infectivity of the COVID-19 virus. Cell.

[CR17] Kretzschmar M, Wallinga J. Mathematical models in infectious disease epidemiology. Modern Infectious Disease Epidemiology. 2009;209–21. 10.1007/978-0-387-93835-6_12

[CR18] Le Thanh T, Andreadakis Z, Kumar A, Gómez Román R, Tollefsen S, Saville M, Mayhew S (2020). The COVID-19 vaccine development landscape. Nat Rev Drug Discov.

[CR19] Li L, Guo P, Zhang X, Yu Z, Zhang W, Sun H (2021). SARS-CoV-2 vaccine candidates in rapid development. Hum Vaccin Immunother.

[CR20] Mercado NB, Zahn R, Wegmann F, Loos C, Chandrashekar A, Yu J (2020). Single-shot Ad26 vaccine protects against SARS-CoV-2 in rhesus macaques. Nature.

[CR21] Mukherjee R (2020). Global efforts on vaccines for COVID-19: Since, sooner or later, we all will catch the coronavirus. J Biosci.

[CR22] Munoz FM, Cramer JP, Dekker CL, Dudley MZ, Graham BS, Gurwith M (2021). Vaccine-associated enhanced disease: case definition and guidelines for data collection, analysis, and presentation of immunization safety data. Vaccine.

[CR23] Noor R (2020). Antiviral drugs against severe acute respiratory syndrome coronavirus 2 infection triggering the coronavirus disease-19 pandemic. Tzu Chi Med J.

[CR24] Noor R (2021). A comparative review of pathogenesis and host innate immunity evasion strategies among the severe acute respiratory syndrome coronavirus 2 (SARS-CoV-2), severe acute respiratory syndrome coronavirus (SARS-CoV) and the Middle East respiratory syndrome coronavirus (MERS-CoV). Arch Microbiol.

[CR25] Noor R (2021). A review on the effectivity of the current COVID-19 drugs and vaccines: are they really working against the severe acute respiratory syndrome coronavirus 2 (SARS-CoV-2) variants?. Curr Clin Micro Rpt.

[CR26] Noor R (2021). Developmental status of the potential vaccines for the mitigation of the COVID-19 pandemic and a focus on the effectiveness of the pfizer-BioNTech and Moderna mRNA vaccines. Curr Clin Microbiol Rep.

[CR27] Noor R, Maniha SM (2020). A brief outline of respiratory viral disease outbreaks: 1889—till date on the public health perspectives. VirusDis.

[CR28] Polack FP, Thomas SJ, Kitchin N, Absalon J, Gurtman A, Lockhart S (2020). Safety and efficacy of the BNT162b2 mRNA Covid-19 vaccine. N Engl J Med.

[CR29] Rai RK, Khajanchi S, Tiwari PK, Venturino E, Misra AK (2021) Impact of social media advertisements on the transmission dynamics of COVID-19 pandemic in India. J Appl Math Comput. 10.1007/s12190-021-01507-y 10.1007/s12190-021-01507-y PMC7910777 33679275

[CR30] Samui P, Mondal J, Khajanchi S (2020). A mathematical model for COVID-19 transmission dynamics with a case study of India. Chaos Solitons Fractals.

[CR31] Sarkar K, Khajanchi S, Nieto JJ (2020). Modeling and forecasting the COVID-19 pandemic in India. Chaos Solitons Fractals.

[CR32] Smatti MK, Al Thani AA, Yassine HM (2018). Viral-induced enhanced disease illness. Front Microbiol.

[CR33] Tseng CT, Sbrana E, Iwata-Yoshikawa N, Newman PC, Garron T, Atmar RL et al (2012) Immunization with SARS coronavirus vaccines leads to pulmonary immunopathology on challenge with the SARS virus. PLOS ONE 7(4):e35421. 10.1371/journal.pone.0035421. Epub 2012 Apr 20. Erratum in: PLOS ONE. 2012;7(8). 10.1371/annotation/2965cfae-b77d-4014-8b7b-236e01a35492 10.1371/journal.pone.0035421 PMC3335060 22536382

[CR34] Ura T, Yamashita A, Mizuki N, Okuda K, Shimada M (2021). New vaccine production platforms used in developing SARS-CoV-2 vaccine candidates. Vaccine.

[CR35] van Dorp L, Acman M, Richard D, Shaw LP, Ford CE, Ormond L, Owen CJ, Pang J, Tan CCS, Boshier FAT, Ortiz AT, Balloux F (2020). Emergence of genomic diversity and recurrent mutations in SARS-CoV-2. Infect Genet Evol.

[CR36] Wadman M, Cohen J (2021) Novavax vaccine delivers 89 % efficacy against COVID-19 in U.K.—but is less potent in South Africa. Science 10.1126/science.abg8101

[CR37] World Health Organization (WHO) (2022) Coronavirus diseases (COVID-19) Dashboard. Updated on 6:03pm CET, 14 January 2022. https://covid19.who.int/ Accessed on 15 January 2022

[CR38] Zellweger RM, Wartel TA, Marks F, Song M, Kim JH (2020). Vaccination against SARS-CoV-2 and disease enhancement: knowns and unknowns. Expert Rev Vaccines.

[CR39] Zheng Y, Zhang Q, Ali A, Li K, Shao N, Zhou X (2021). Sustainability of SARS-CoV-2 induced humoral immune responses in COVID-19 patients from hospitalization to convalescence over six months. Virol Sin.

